# Primary Board Appointed for the Purpose of Examining Young Men before Entering the Study of Medicine

**Published:** 1872-01

**Authors:** 


### Correspondence.

At the 22d Annual Meeting of the National Medical Association the following resolution was adopted:

*Resolved*, That each State and Local Medical Society be requested to provide, as a permanent part of its organization, a Board of Censors for determining the educational qualifications of such young men as propose to commence the study of medicine, and that no member of such Societies be permitted to receive a student into his office until such student presents a certificate of proper preliminary education from the Censors appointed for that purpose, or a degree from some literary college of known good standing.

The necessity of enforcing this regulation is too well appreciated by every member of the profession to require extended notice.

The Primary Board appointed at the last annual meeting of Erie County Medical Society, held January 9th, 1872, for the purpose of examining young men preparatory to the study of medicine, would announce that persons requiring such examination will be expected to furnish evidence of good moral character, and to show a satisfactory acquaintance with the following subjects:

The rules of the construction of English Language, English Literature, Geography, History, (Ancient and Modern,) with Arithmetic, Algebra to Quadratics, Elementary Geometry, Moral Science, with Elementary Chemistry and Natural Philosophy, or to show a diploma from some recognized literary college.

In this attempt to impress the importance of good preliminary education upon young men about to enter upon the study of medicine we hope to have your support and co-operation.

The Board will meet for the examination of applicants on the last Wednesday of every month at the office of Dr. H. R. Hopkins, No. 9 West Swan street, at 11 o'clock A. M.

M. B. Folwell,
H. R. Hopkins,
M. Willoughby, Jr.

Buffalo, January 17, 1872. Primary Board.

